# Supplementary material for: Hypoxia and loss of PHD2 inactivate stromal fibroblasts to decrease tumour stiffness and metastasis
Source: EMBO Rep. 2015 Aug 31;16(10):1394–408. doi: 10.15252/embr.201540107 (PMC4662858; doi:10.15252/embr.201540107)
Supplement: Supplementary file 1 — Expanded View Figures PDF [file EMBR-16-1394-s001.pdf]

Expanded View Figures

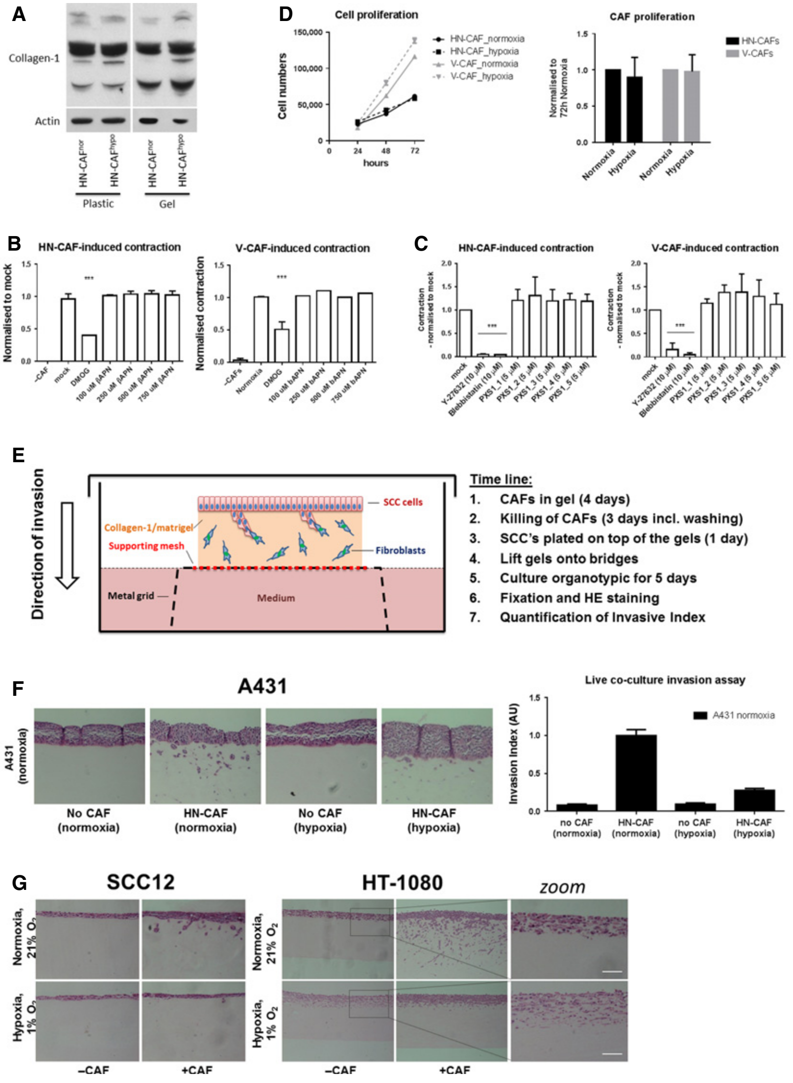

Figure EV1. Hypoxia suppresses CAF-induced contraction and invasion.

A Immunoblotting analysis of collagen I.

B, C Bars shows quantification of HN-CAF- and V-CAF-induced contraction relative to mock treatment. Bars represent mean ± s.d. n = individual cells from 3 experimental repeats. ***$P < 0.001$; unpaired Student's t-test (two-tailed).

D Proliferation assay of CAFs. Left graph shows mean ± s.d. growth over time. The graph shows a representative experiment done in triplicate. Bars represent mean ± s.d. growth after 72-h incubation. n = 4 independent experimental repeats.

E Schematic representation including time line of the organotypic co-culture system used for studying CAF-induced SCC invasion.

F, G HN-CAF-induced cancer cell invasion. (F) H&E-stained sections of A431 cells co-cultured in an organotypic system in the presence of HN-CAFs. Bars shows mean ± s.d. invasive index of A431 cancer cell invasion. n = 5 experimental points from 2 independent experiments. (G) H&E-stained sections of SCC12 and HT-1080 co-cultured in an organotypic system in the presence of HN-CAFs. Zoomed images demonstrate that HT-1080 cell alone induces invasion in hypoxia. Scale bar: 100 μm.

 © 2015 The Authors

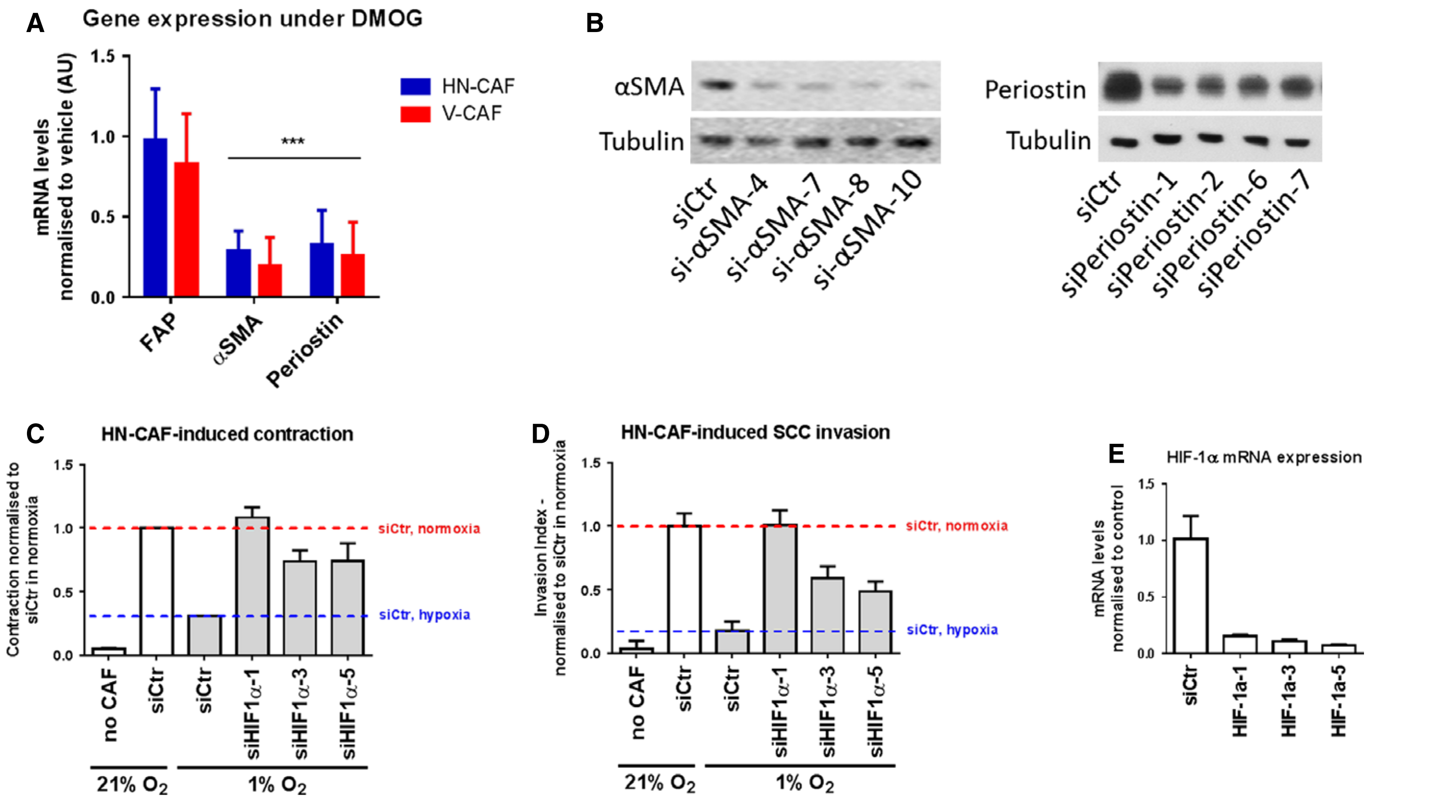

Figure EV2. Hypoxia suppresses CAF activation.

A qPCR analysis of gene expression levels of various CAF activation markers after 72 h of DMOG treatment. Bars show mean ± s.d. mRNA levels quantified by qPCR and normalised to vehicle treatment (DMSO). ***$P < 0.001$, unpaired Student's *t*-test (two-tailed).

B Validation of siRNA knock-down of αSMA (left panel) and periostin (right panel) by Western blotting.

C, D Depletion of HIF-1α using multiple siRNAs rescues loss of HN-CAF-induced contraction and invasion observed in hypoxia. Bars show quantification of HN-CAF-induced contraction (C) and HN-CAF-induced SCC12 cancer cell invasion (D). Bars show mean ± s.d. $n = 2$ experimental repeats.

E Validation of siRNA knock-downs of HIF-1α by qPCR. Bars show mean ± s.d. $n = 3$ experimental repeats.

© 2015 The Authors

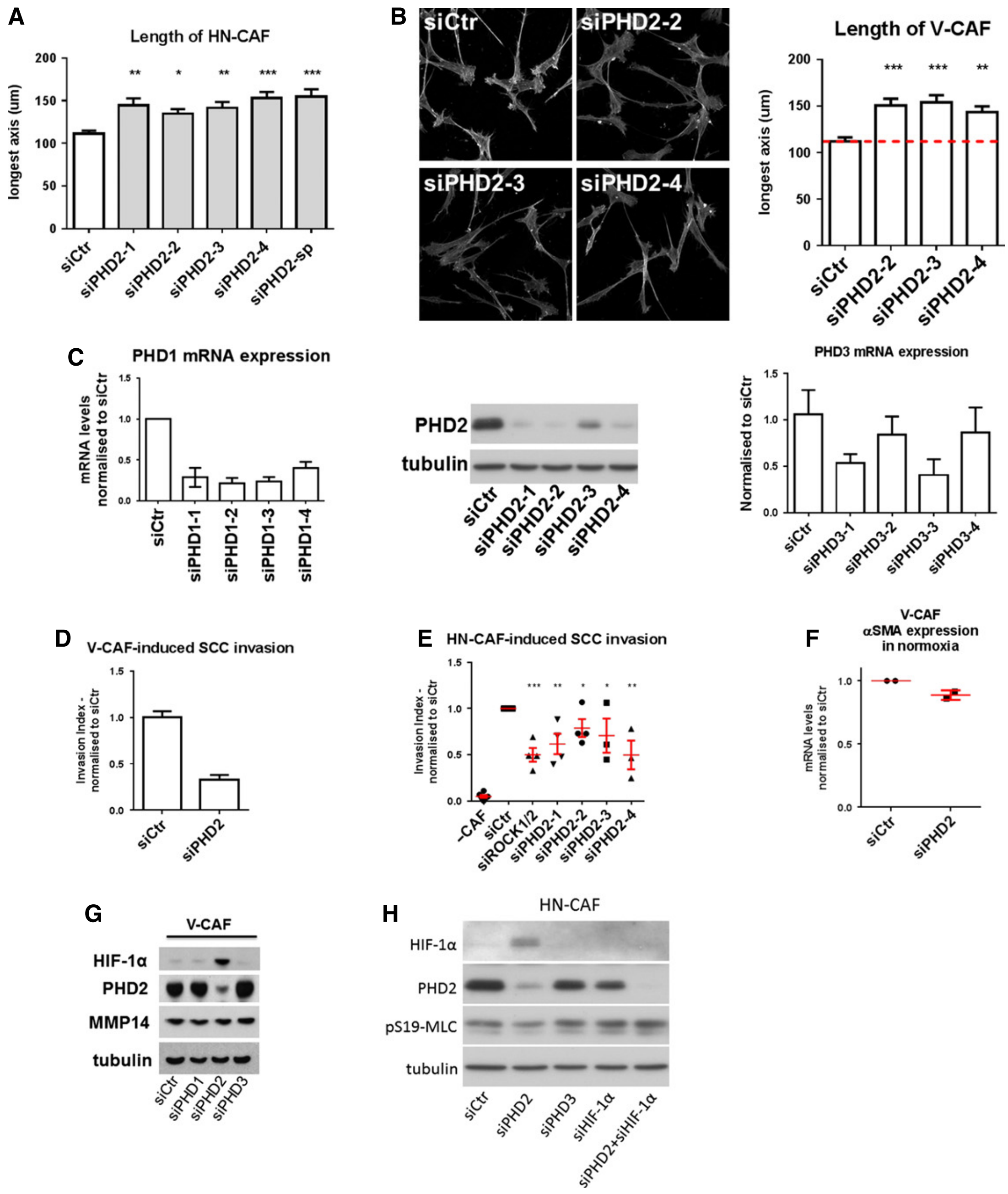

Figure EV3. Loss of PHD2 suppresses CAF-induced matrix remodelling and invasion.

A Bars show quantification of the longest distance of HN-CAFs plated in 3D collagen I/Matrigels. Bars indicate mean ± s.d. *$P < 0.05$; **$P < 0.01$; ***$P < 0.001$; one-way ANOVA test. $n = 3$ experimental repeats.

B F-actin staining of V-CAFs grown on top of collagen I/Matrigels. Bars show quantification of the longest distance of V-CAFs. Bars indicate mean ± s.d. **$P < 0.01$; ***$P < 0.001$; one-way ANOVA test. $n = 2$ experimental repeats.

C Validation of multiple siRNAs targeting PHD2 by Western blotting. Validation of multiple siRNAs targeting PHD1&3 by qPCR. Bars show mean ± s.d. $n = 2$ independent experimental repeats done in triplicate.

D Depletion of PHD2 decreases V-CAF-induced invasion. Bars show mean ± s.d. invasive index. $n = 2$ experimental repeats.

E Depletion of PHD2 using multiple siRNAs decreases HN-CAF-induced invasion. Bars show mean ± s.d. invasive index. $n > 3$ experimental repeats. *$P < 0.05$; **$P < 0.01$; ***$P < 0.001$; unpaired Student's t-test (two-tailed).

F PHD2 regulates αSMA mRNA levels. V-CAFs were siRNA-depleted for PHD2, and mRNA levels of αSMA were quantified by qPCR 72 h post-transfection. CAFs were plated on gels. Each data point represents an independent experiment done in triplicate. Line and error bars indicate mean ± s.d.

G Immunoblotting analyses of the HIF-1α, PHD2 and MMP14. siRNA-depleted V-CAFs were grown on gels for 72 h post-transfection.

H Immunoblotting validation of the rescue experiment presented in Fig 5G.

 © 2015 The Authors

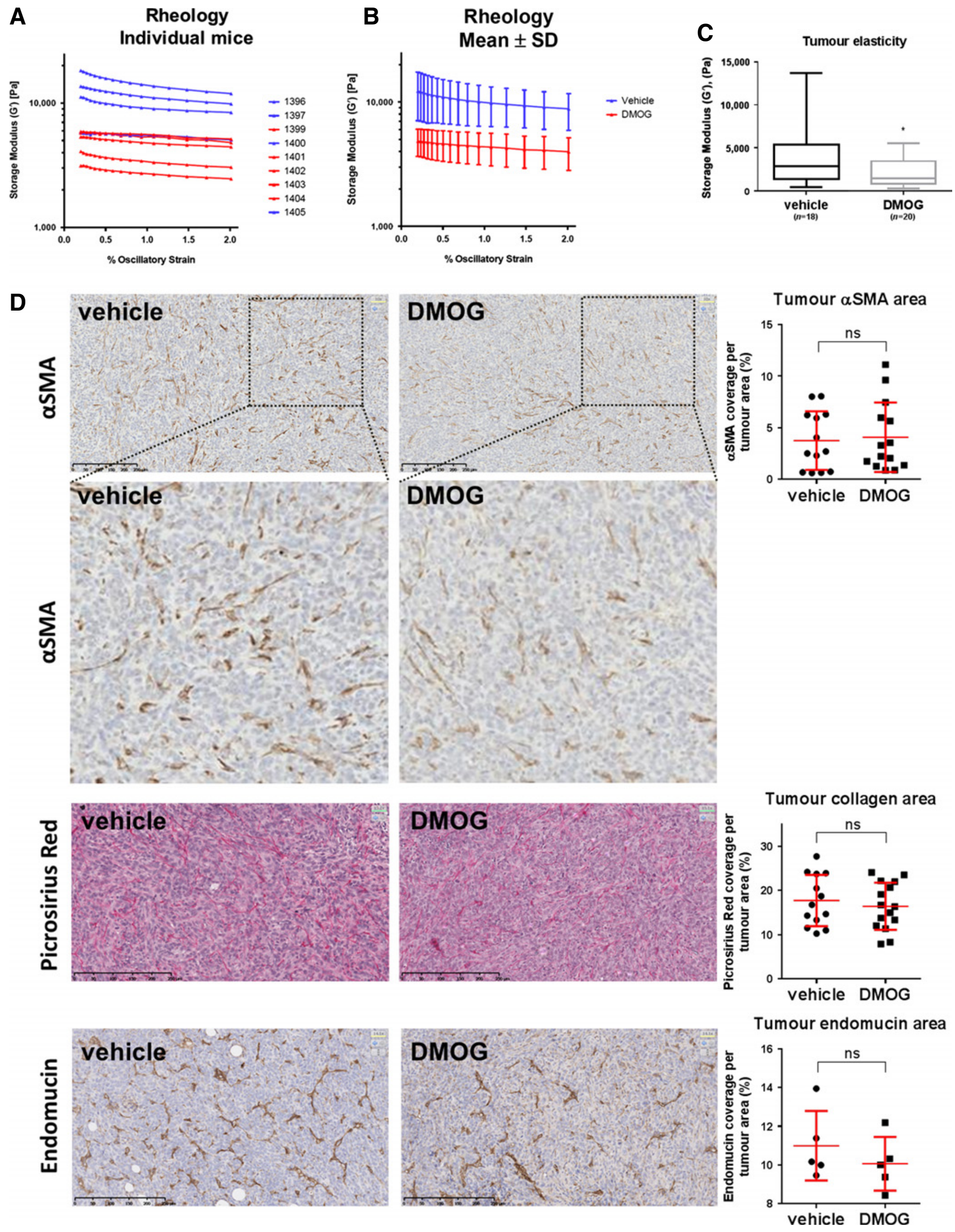

Figure EV4. DMOG treatment reduces tumour stiffness and metastasis.

A, B Tumour stiffness. 4T1 cells were orthotopically injected into the fat pad of 8-week-old female BALB/c mice. After 1 week, the mice were injected i.p. with 8 mg DMOG every second day. The mammary tumours were excised after 3 weeks of DMOG treatment. Shear rheology was performed immediately on fresh mammary tumour tissue. (A) The graph shows each individual mouse from one independent experiment. Vehicle (PBS)-treated mice are shown in blue and DMOG-treated in red. (B) The graph shows the mean ± s.d. of the same experiment. Storage modulus was always measured over a decade of strain from 0.2% to 2% at a fixed angular frequency of 0.5 rad/s and a temperature of 21°C.

C Whisker plot shows storage modulus of freshly excised primary breast tumour. Number (*n*) of mice is indicated. Box and whiskers graph: line = median, box = distribution of 50% of values, whiskers = minimum to maximum. *$P < 0.05$; unpaired Student's *t*-test (two-tailed).

D IHC stainings of paraffin-embedded tumour sections. Sections were stained for αSMA, picrosirius red (collagen I and III) and endomucin (blood vessels). Scatter plots show quantification of percentage of cell area covered in the section. Each data point represents one mouse. Line and error bars indicate mean ± s.d. ns, non-significant; unpaired Student's *t*-test (two-tailed).

© 2015 The Authors

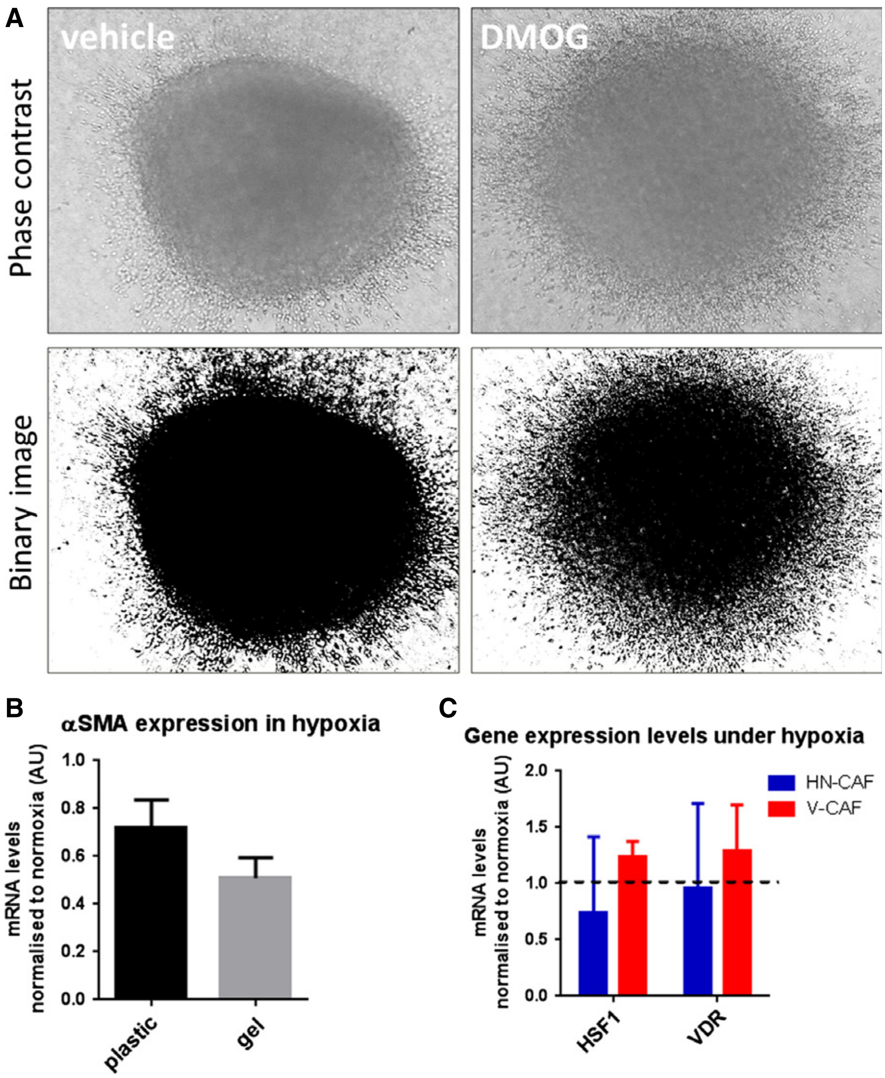

Figure EV5. 4T1 spheroid invasion.

A 4T1 spheroids were allowed to invade into a 3D collagen I/Matrigel for 72 h. Phase contrast images and binarised images are shown.

B αSMA mRNA levels after incubation on plastic and gels for 72 h in hypoxia. Bars show mean ± s.d. mRNA levels normalised to normoxic conditions. $n > 3$ experimental repeats.

C HSF1 and VDR mRNA levels 72 h in hypoxia. Bars show mean ± s.d. mRNA levels normalised to normoxic conditions. $n = 3$ experimental repeats.

 © 2015 The Authors
